# Social Downsizing Stress (SDS): A novel paradigm for studying social transitions in mice

**DOI:** 10.1016/j.ibneur.2026.10.003

**Published:** 2026-10-25

**Authors:** Yoshihiro Tanaka, Hiroshi Ueno, Eriko Kitano, Yu Takahashi, Sachiko Mori, Shinji Murakami, Kenta Wani, Yosuke Matsumoto, Motoi Okamoto, Takeshi Ishihara

**Affiliations:** a Department of Psychiatry, Kawasaki Medical School, Kurashiki, 701-0192, Japan; b Department of Medical Technology, Kawasaki University of Medical Welfare, Okayama, 701-0193, Japan; c Department of Neuropsychiatry, Graduate School of Medicine, Dentistry and Pharmaceutical Sciences, Okayama University, Okayama, 700-8558, Japan; d Department of Medical Technology, Graduate School of Health Sciences, Okayama University, Okayama, 700-8558, Japan

**Keywords:** Social downsizing stress, Group size reduction, Resocialization, Stress coping, Mice

## Abstract

**Background:**

Shrinking social networks and the transition to nuclear families represent significant structural changes in modern society. While traditional animal models have focused on total social isolation or defeat, the behavioral consequences of a dynamic reduction in group size remain poorly understood. We established a novel "Social Downsizing Stress (SDS)" model to investigate how a transition from high-density to low-density housing affects behavioral phenotypes and whether these effects are reversible through resocialization.

**Methods:**

Mice were assigned to four groups: Stable-6 (S6), Stable-3 (S3), SDS (6–3 mice at 8 weeks), and Resocialization (ReSDS; 6–3 at 8 weeks, followed by 3–6 at 11 weeks). Behavioral batteries were conducted at 10 weeks (acute phase) and 13–14 weeks (chronic/recovery phase).

**Results:**

At 10 weeks, the SDS group exhibited a unique hyper-reactive phenotype distinct from the S3 group, characterized by significantly increased locomotor activity in the open field, increased entries in the light/dark transition test, and reduced social resting time. Both the S3 and SDS groups showed impaired spatial working memory in the Y-maze. By 13–14 weeks, resocialization (ReSDS) successfully restored social resting behavior and cognitive performance to control levels. However, SDS-induced hyperactivity in the open field persisted in both the SDS and ReSDS groups, regardless of the subsequent return to a higher-density environment. Furthermore, the ReSDS group exhibited significantly lower immobility in the tail suspension and forced swim tests compared to the S3 group.

**Conclusion:**

Social downsizing induces a distinct behavioral phenotype that differs from chronic low-density housing. While social interaction and cognitive functions retain high plasticity, hyperactivity persisted throughout the tested period (up to 13–14 weeks) despite resocialization, suggesting a long-lasting, though not yet confirmed irreversible, consequence of losing social density. This model provides a new framework for investigating the enduring neurobehavioral impacts of shrinking social environments on mental health.

## Introduction

Recent decades have witnessed structural changes in the social environment, including the rise of nuclear families and reductions in household size. In human society, it has been suggested that a decrease in the number of cohabitants and the shrinking of social networks is associated with adverse outcomes in social-cognitive development, mental health, and stress responses (Cacioppo and Hawkley, 2009, Perner et al., 1994, Tooley et al., 2021). These human phenomena are shaped by complex emotional, cognitive, cultural, and economic factors that cannot be directly captured in a rodent model; the present study is not intended to model these factors, but rather to isolate one structural dimension common to many such situations — a quantitative reduction in the number of familiar social partners — under controlled experimental conditions. Conversely, animal research has predominantly utilized robust stress models involving total social deprivation or direct aggression, such as social isolation or social defeat (Krishnan et al., 2007). However, the impact of situations where social contact is maintained but the group size (housing density) alone is reduced on an individual’s behavior and stress response remains largely unexamined.

Many established social stress models focus on social isolation through individual housing or specific, potent stressors such as social defeat and chronic restraint (Koolhaas et al., 2011). While these models are highly effective in reproducing the "disappearance" or "infringement" of social relationships, they do not directly mimic more common and gradual environmental changes, such as the reduction of a social group’s scale (Jarcho et al., 2016). Consequently, the differences in impact between an environment maintained at a constant low density and the dynamic process of transitioning from high to low density (Social Downsizing) have not been sufficiently verified, nor has the potential reversibility of these effects upon returning to a larger social group.

The scale of a social group is a critical factor defining the frequency of social interactions, hierarchical structures, the volume of stimuli, and environmental predictability (McEwen, 2007). A sudden reduction in group size can trigger a reorganization of existing social relationships and hierarchies, potentially lowering environmental stability (Jarcho et al., 2016). Based on broader stress and social-buffering frameworks, we hypothesize that such shifts in social scale could engage stress-responsive systems — for example, activation of the hypothalamic–pituitary–adrenal (HPA) axis, modulation of prefrontal cortex-dependent cognitive functions, and altered stress-coping styles (Arnsten, 2009) — although these mechanisms have not been directly examined in the SDS paradigm. Specifically, a reduction in a familiar group size might be perceived as a signal of compromised environmental safety, potentially leading to hyper-vigilance or altered arousal (Coan and Sbarra, 2015) this interpretation remains speculative in the absence of direct physiological or neurobiological measurements. Social interaction is more generally known to serve as a "social buffering" mechanism that mitigates stress responses (Hennessy et al., 2009); by extension, we hypothesize that a reduction in social scale may weaken this buffering effect, although this specific prediction has not yet been empirically tested. Nevertheless, the behavioral consequences of reducing group size while maintaining social contact have not been systematically investigated.

In this study, we established a novel experimental paradigm in which mice were housed at six per cage after weaning and then transitioned to three per cage at 8 weeks of age (postnatal day [PND] 56). We selected this timing because, in C57BL/6 mice, the period around PND 56 is generally regarded as the boundary between late adolescence and young adulthood, following the completion of major pubertal and adolescent developmental milestones (Laviola et al., 2003, Spear, 2000 Jun) but preceding full behavioral and physiological maturity. This timing allowed animals to first establish stable social relationships within a consistent six-mouse group from weaning (PND 21) through early adolescence (approximately 5 weeks), before the downsizing manipulation was introduced at a developmentally later, more stable stage. We reasoned that this design would minimize confounding by ongoing adolescent developmental change while still capturing a transition broadly analogous to human social-network contraction occurring around the transition to independent living. This timing is also consistent with our own prior work using a related developmental group-size transition paradigm, in which mice were similarly transitioned from a larger to a smaller cage-mate number at 8 weeks of age (Tanaka et al., 2026). Whereas that previous study initiated behavioral testing approximately one week after the group-size transition, in the present study we extended this interval to approximately two weeks before the first behavioral assessment, in order to allow the reduced-density social environment to fully stabilize and to ensure that the observed acute-phase phenotype reflected a sustained, rather than a transient, response to the transition. We defined this paradigm as "Social Downsizing Stress (SDS)". This model is characterized by manipulating only the dynamic reduction of social scale without accompanying total social isolation. Through a battery of behavioral tests, we comprehensively examined (1) acute behavioral changes immediately following the downsizing, (2) behavioral reorganization in the chronic phase over time, and (3) the presence of reversibility through Resocialization (ReSDS).

This study presents a new framework for understanding changes in social scale as a stress factor in its own right, rather than focusing solely on social isolation. By demonstrating that social downsizing can induce transient cognitive modulation and persistent changes in stress-coping styles, we aim to deepen the understanding of behavioral adaptation mechanisms to dynamic social changes (Rook, 2013). This model serves as a valuable experimental foundation for analyzing neurobehavioral responses to fluctuations in social structure, providing insights into the long-lasting behavioral consequences that shrinking social environments may leave behind.

## Experimental procedures

### Ethics statements

All animal experiments were conducted in accordance with the Guidelines for the Care and Use of Laboratory Animals of the National Institutes of Health (NIH, USA) and complied with the ARRIVE 2.0 guidelines (https://www.nc3rs.org.uk/arrive-guidelines). The study was approved by the Committee for Animal Experiments of Kawasaki Medical School. All efforts were made to minimize animal suffering and to reduce the number of animals used. Mice were monitored daily for health and welfare, and no unexpected adverse events occurred during the study. Humane endpoints were predefined and applied when necessary.

### Animals

Male C57BL/6 N mice were obtained from Jackson Laboratory Japan (Tokyo, Japan) at postnatal day (PND) 21. Only male mice were used to avoid potential variability associated with the estrous cycle. Upon arrival, animals were randomly assigned to experimental groups. To minimize litter effects, mice from multiple litters were mixed and evenly distributed across groups. Animals were housed under standard laboratory conditions (temperature 23–26 °C; humidity 50–60%; 12-h light/dark cycle, lights on at 08:00) with ad libitum access to standard chow and water. Mice were maintained in standard polycarbonate cages (220 × 340 × 150 mm) with nesting materials. No environmental enrichment was provided throughout the study. Bedding was changed once per week. Behavioral testing was conducted during the light phase between 09:00 and 16:00. Mice were allowed to acclimate to the animal facility for at least 3 days before the initiation of experimental manipulation. The total number of animals used in this study was 40 (n = 10 per group). Sample size was determined based on previous behavioral studies and pilot data. To minimize positional variability within the housing environment, cages from all experimental groups were placed on racks at the same height. All experimental groups were tested in parallel rather than sequentially. Body weight gain was calculated as a percentage using the following formula: (body weight at postnatal week 10 / body weight at postnatal week 8) × 100.

### Housing and social downsizing stress (SDS) procedure

At postnatal day (PND) 21, mice were randomly assigned to one of four housing conditions: Stable-6 (S6), Stable-3 (S3), Social Downsizing Stress (SDS), or Resocialization (ReSDS) (Fig. 1**A**). In the S6 group, mice were housed continuously in groups of six per cage from PND 21 until the end of the experiment. In the S3 group, mice were housed continuously in groups of three per cage throughout the study. These groups served as stable-density reference conditions. In the SDS group, mice were initially housed in groups of six per cage from PND 21 to PND 56 (8 weeks of age). At PND 56, group size was reduced from six to three mice per cage (6→3), and mice remained in this reduced group size thereafter. This reduction in social group size without complete social isolation was defined as Social Downsizing Stress (SDS). In the Resocialization (ReSDS) group, mice underwent the same downsizing procedure (6→3 at PND 56) and were maintained in groups of three for three weeks. At PND 77 (11 weeks of age), group size was restored from three to six mice per cage (3→6), and mice remained in groups of six until the completion of behavioral testing (Fig. 1**B**). When downsizing was performed at PND 56, each original six-mouse cage was randomly divided into two subgroups of three mice. Thus, each reduced cage (three mice per cage) consisted exclusively of mice that had previously been housed together. No mixing between different original cages occurred during the downsizing procedure. For the resocialization procedure (3→6 at PND 77), mice were reunited with their original cage-mates, thereby restoring the initial six-mouse social configuration. All cages were maintained under identical environmental conditions throughout the experiment. To minimize potential rack-position effects, cage locations were rotated weekly.

**Fig. 1:**
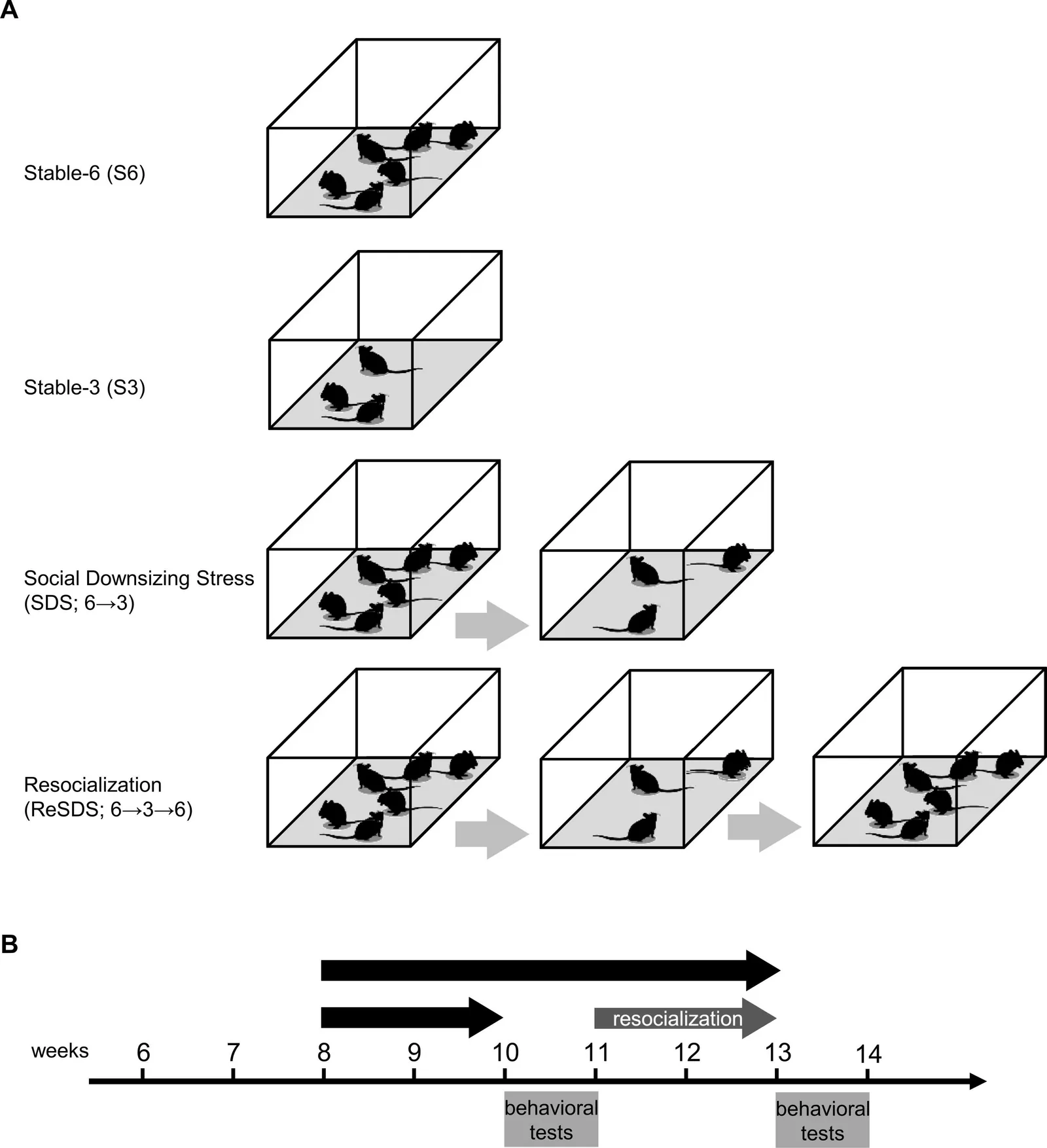
Experimental design of the Social Downsizing Stress (SDS) paradigm. (A) Schematic illustration of the experimental timeline and housing conditions. Male mice were housed from weaning (postnatal day 21) under one of four conditions: Stable-6 (S6; six mice per cage throughout), Stable-3 (S3; three mice per cage throughout), Social Downsizing Stress (SDS; six mice per cage until 8 weeks of age followed by reduction to three mice per cage, 6→3), and Resocialization (ReSDS; 6→3→6). Downsizing was performed at postnatal day 56, and resocialization was conducted at postnatal day 77 by reuniting mice with their original cage-mates. (B) Schedule of behavioral testing. Behavioral assessments were conducted during two phases: an early phase at 10 weeks of age to evaluate acute effects of social downsizing, and a later phase at 13–14 weeks of age to assess long-term effects and the impact of resocialization.

### Behavioural tests

All behavioral tests were conducted in a dedicated behavioral testing room between 09:00 and 16:00 during the light phase of the light–dark cycle. Beginning two days before the first day of testing at each time point, mice were habituated to the testing room by being placed in the room for 2 h per day, without exposure to any testing apparatus, in order to reduce novelty-induced stress associated with the testing environment itself. To minimize potential confounding effects of olfactory cues, all apparatuses and objects used in the tests were thoroughly cleaned with 70% ethanol followed by hypochlorous acid solution after each trial. Behavioral assessments were performed using experimentally naïve mice at each time point (i.e., animals that had not been exposed to any behavioral tasks prior to the first day of testing at that time point). One behavioral test was performed per day, with a 24-h interval between consecutive tests. Tests were administered in the following fixed order: grip-strength test, hot plate test, light/dark transition test, open field test, Y-maze test, and social interaction test — performed at both 10 weeks and 13–14 weeks of age — followed additionally by the tail-suspension test and the Porsolt forced-swim test, in that order, only at 13–14 weeks of age. This order was chosen to minimize carryover effects by performing less stressful, non-aversive tasks (e.g., grip strength) before more aversive or stress-inducing tasks (e.g., open field, forced swim). Accordingly, the full battery spanned 6 consecutive days at 10 weeks of age and 8 consecutive days at 13–14 weeks of age. All behavioral scoring and analyses were conducted by trained experimenters who were blinded to the experimental conditions.

### Grip-strength test

Neuromuscular strength was assessed using a forelimb grip-strength test. Grip strength was measured with a grip dynamometer. Mice were allowed to grasp a horizontal metal grid with their forepaws while being gently pulled backward by the tail in a horizontal plane until the grip was released. The peak force exerted before release was automatically recorded in centinewtons (cN). Each mouse was tested in five consecutive trials with an inter-trial interval of at least 30 s to prevent fatigue. The highest value obtained across trials was used for statistical analysis. All measurements were performed by an experimenter blinded to the experimental conditions.

### Hotplate test

Nociceptive sensitivity was assessed using the hot plate test. Each mouse was placed individually on the heated surface enclosed by a transparent acrylic cylinder to prevent escape. Latency to the first nociceptive response (hind paw licking, hind paw shaking, or jumping) was recorded with a cutoff time of 30 s to prevent tissue damage. If no response occurred within the cutoff period, the mouse was immediately removed, and the maximum latency (30 s) was assigned for analysis. Each mouse was tested once to avoid habituation or sensitization effects. All assessments were conducted by an experimenter blinded to the experimental conditions.

### Light/dark transition test

The light/dark transition test was used to assess anxiety-related behavior and exploratory activity. The apparatus consisted of an acrylic box (22 × 44 × 40 cm) divided into two compartments by a partition with a small opening. One compartment had white acrylic walls and was brightly illuminated at approximately 200 lux, whereas the other had black acrylic walls and was dimly illuminated at approximately 50 lux. Both compartments had white plastic floors. At the beginning of each trial, a mouse was placed in the dark compartment and allowed to freely explore both compartments for 6 min with the opening unobstructed. Behavioral data were recorded using ANY-maze software (Stoelting Co., Wood Dale, IL, USA), including total distance traveled, number of transitions between compartments, time spent in the light compartment, and latency to first entry into the light compartment.

### Open field test

The open field test was used to assess locomotor activity and anxiety-related behavior. The apparatus consisted of a square acrylic arena (45 × 45 × 40 cm) with opaque walls and a white plastic floor. The arena was evenly illuminated at approximately 100 lux. At the beginning of each trial, a mouse was placed gently in the center of the arena and allowed to freely explore for 20 min. Behavioral data were recorded using ANY-maze software (Stoelting Co., Wood Dale, IL, USA), including total distance traveled, number of entries, and time spent in the central area. The central area was defined as the inner 20 × 20 cm zone of the arena. Total distance traveled was used as an index of locomotor activity, whereas time spent in the central area was interpreted as an indicator of anxiety-related behavior.

### Y-maze test

The Y-maze test was used to assess spatial working memory based on spontaneous alternation behavior. The apparatus consisted of a Y-shaped maze with three identical arms (arm length, 40 cm; bottom arm width, 3 cm; upper arm width, 10 cm; wall height, 12 cm) positioned at 120° angles from each other. The maze was evenly illuminated at approximately 100 lux. At the beginning of each trial, a mouse was placed at the end of one arm and allowed to freely explore the maze for 6 min. An arm entry was defined as all four paws entering an arm. Spontaneous alternation was defined as consecutive entries into three different arms (e.g., ABC, BCA). Visual cues were placed around the testing chamber and remained visible throughout the test. Mice were tested without prior exposure or habituation to the maze. Behavioral data were recorded and analyzed using ANY-maze software, including the total distance traveled, the number of arm entries, and the number of alternations, from which the alternation percentage was calculated. The alternation percentage was calculated using the following formula: Alternation (%) = [Number of alternations / (Total number of arm entries − 2)] × 100.

### Social interaction test in a novel environment

Social behavior was assessed by allowing two unfamiliar mice to freely interact in a novel environment. Two mice from the same experimental group, housed in separate cages (and therefore unfamiliar with one another prior to testing), were placed together in a square arena (45 × 45 × 40 cm) and allowed to interact freely for 10 min. Behavioral recordings were analyzed using SMART software (version 3.0; Panlab, Harvard Apparatus, Barcelona, Spain). The following parameters were quantified: total distance traveled, total number of contacts, and mean duration per contact.

### Tail-suspension test

Depressive-like behavior was evaluated using the tail-suspension test. Each mouse was suspended by the tail in a white plastic chamber positioned 60 cm above the floor, using adhesive tape placed approximately 1 cm from the tip of the tail. Behavior was recorded for 6 min using a video camera, and immobility time was subsequently quantified. Immobility was defined as the absence of active escape-directed movements, during which the mouse hung passively without struggling for at least 1 s. Data acquisition and analysis were performed using ANY-maze software (Stoelting Co., Wood Dale, IL, USA).

### Porsolt forced-swim test

The Porsolt forced-swim test was used to assess depressive-like behavior. The apparatus consisted of four Plexiglas cylinders (20 cm in height × 10 cm in diameter) filled with water at 23 °C to a depth of 7.5 cm, in accordance with previously described procedures. Each mouse was placed individually into a cylinder and allowed to swim for 6 min, during which behavior was recorded. Immobility time was quantified using ANY-maze software, consistent with the criteria applied in the tail suspension test.

### Statistical analysis

All statistical analyses were performed using GraphPad Prism (GraphPad Software, San Diego, CA, USA). Data were analyzed using one-way analysis of variance (ANOVA) followed by Tukey’s post hoc test for multiple comparisons. The significance level was set at *p* < 0.05. Data are presented as box-and-whisker plots, with the center line representing the median, the box representing the interquartile range, and the whiskers representing the minimum and maximum values. F-values and p-values from the one-way ANOVA for all major behavioral outcomes are summarized in Table S1.

## Results

### Physical characteristics and sensory response at 10 weeks of age

At 10 weeks of age, we assessed the physical characteristics and basic sensory-motor functions of mice in the S6, S3, and SDS groups. There were no significant differences in body weight among the three experimental groups (Fig. 2**A**). Similarly, the percentage of body weight gain from 8 to 10 weeks of age showed no significant variation regardless of the housing density or the experience of social downsizing (Fig. 2**B**). In terms of physical performance, grip strength was comparable across all groups, with no statistically significant differences observed (Fig. 2**C**). In the hot plate test, which was used to evaluate sensory response, both the S3 and SDS groups exhibited a significantly longer latency to response compared to the S6 control group (Fig. 2**D**). However, no significant difference in latency was found between the S3 and SDS groups (Fig. 2**D**). These results indicate that the intervention of social downsizing stress does not adversely affect general physical growth or basic muscular strength at the acute phase.

**Fig. 2:**
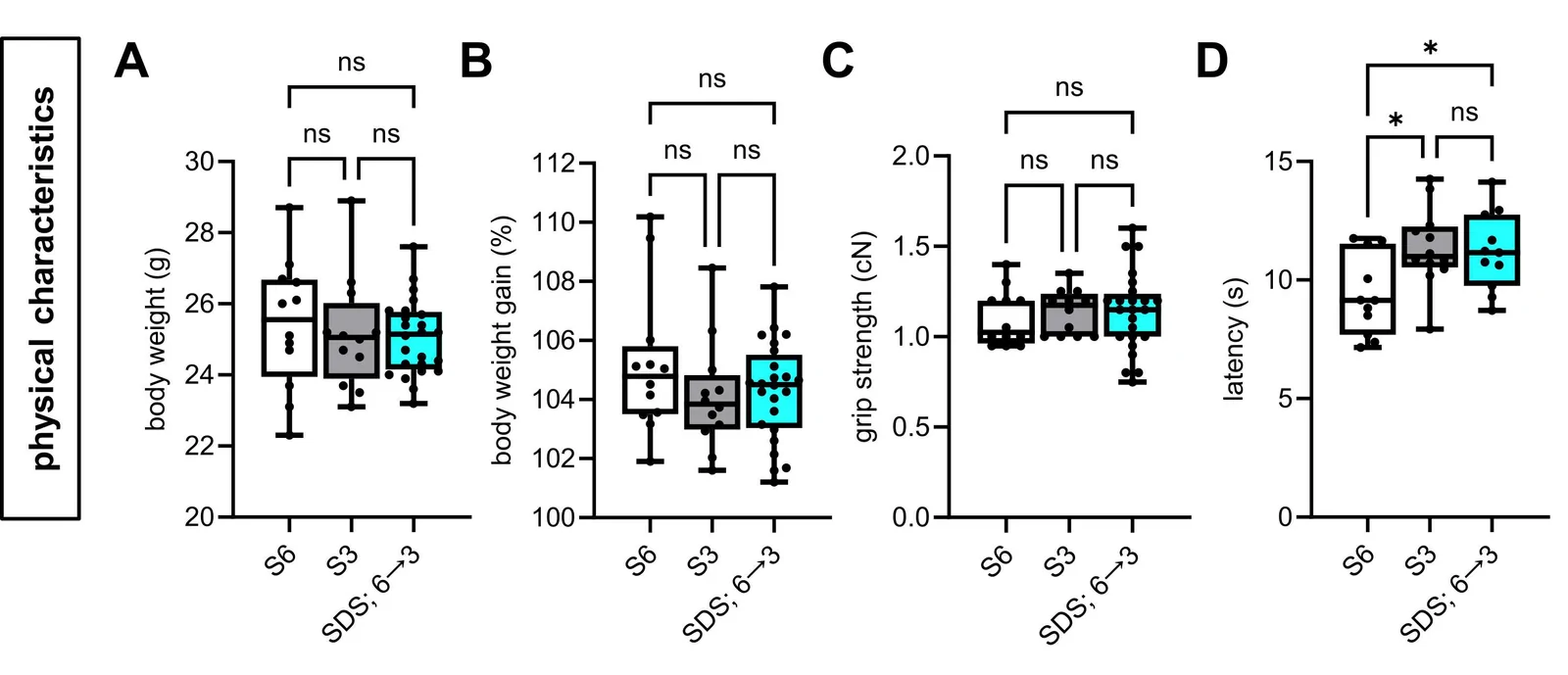
Effects of social downsizing on physical characteristics and sensory response at 10 weeks of age. (A) Body weight at 10 weeks of age. (B) Body weight gain from 8 weeks to 10 weeks of age. (C) Grip strength. (D) Latency to response in the hot plate test. Data are presented as box-and-whisker plots. Statistical significance was determined by one-way ANOVA followed by Tukey’s post-hoc test. *p < 0.05; ns, not significant.

### Behavioral phenotypes at 10 weeks of age

The behavioral consequences of social downsizing stress (SDS) were comprehensively evaluated through a battery of tests at 10 weeks of age. In the light/dark transition test, the SDS group showed a significantly higher number of entries into the light compartment compared to both the S6 and S3 groups (Fig. 3B). However, no significant differences were observed in the total distance traveled (Fig. 3A), time spent in the light area (Fig. 3**C**), or the latency to the first entry into the light area among the groups (Fig. 3**D**).

**Fig. 3:**
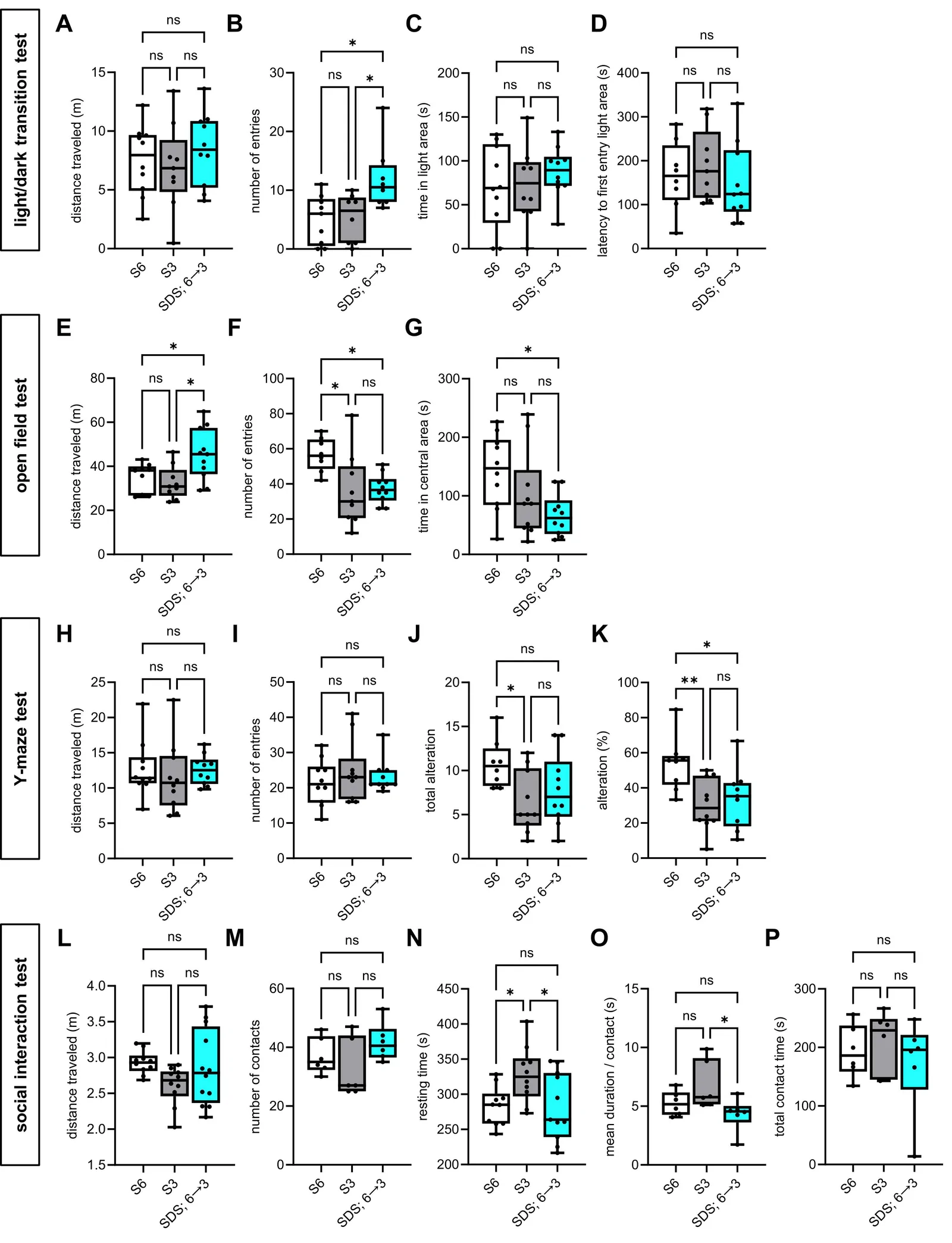
Effects of social downsizing on behavioral phenotypes at 10 weeks of age. (A–D) Light/dark transition test: total distance traveled (A), number of entries into the light compartment (B), time spent in the light area (C), and latency to first entry into the light area (D). (E–G) Open field test: distance traveled (E), number of entries (F), and time spent in the central area (G). (H–K) Y-maze test: distance traveled (H), number of entries (I), total number of spontaneous alternations (J), and percentage of spontaneous alternation (K). (L–P) Social interaction test: distance traveled (L), number of contacts (M), resting time (N), mean duration per contact (O), and total contact time (P). Data are presented as box-and-whisker plots. Statistical significance was determined by one-way ANOVA followed by Tukey’s post-hoc test. *p < 0.05, **p < 0.01; ns, not significant.

In the open field test, the SDS group exhibited a distinct hyperactive phenotype, characterized by a significantly increased distance traveled compared to both S6 and S3 controls (Fig. 3**E**). Furthermore, both the S3 and SDS groups showed a significantly lower number of entries into the central area (Fig. 3**F**) and spent significantly less time in the central area compared to the S6 group (Fig. 3**G**), suggesting an increase in anxiety-like behavior following social density reduction.

Assessment of spatial working memory via the Y-maze test revealed that while locomotor activities, such as distance traveled (Fig. 3**H**) and number of entries (Fig. 3**I**), remained comparable across groups, cognitive performance was impaired. Specifically, the S3 group showed a significant decrease in total alternations compared to the S6 group (Fig. 3**J**). Most notably, both the S3 and SDS groups exhibited a significantly lower percentage of spontaneous alternation compared to S6 (Fig. 3**K**), indicating that a reduction in group size negatively impacts working memory.

In the social interaction test, there were no significant differences in distance traveled (Fig. 3**L**), number of contacts (Fig. 3**M**), or total contact time among the groups (Fig. 3**P**). However, both the S3 and SDS groups exhibited significantly reduced resting time compared to the S6 group (Fig. 3**N**). Additionally, the mean duration per contact was significantly shorter in the SDS group compared to the S3 group (Fig. 3**O**), reflecting a more fragmented social interaction pattern specific to the social downsizing experience.

### Physical characteristics and sensory response at 13 weeks of age

At 13 weeks of age, the physical characteristics and sensory-motor functions were re-evaluated to determine the long-term effects of social downsizing and the subsequent impact of resocialization. Similar to the findings at 10 weeks, no significant differences were observed in body weight among the four experimental groups (Fig. 4**A**). The percentage of body weight gain from 11 to 13 weeks of age also remained stable across all groups, with no statistically significant variations regardless of the social environment transitions (Fig. 4**B**). In the assessment of physical performance, the S3 group exhibited a significantly higher grip strength compared to the S6 group, while no significant differences were detected among the other groups (Fig. 4**C**). In the hot plate test, both the S3 and SDS groups showed significantly increased latencies to response compared to the S6 control group (Fig. 4**D**). Notably, the latency in the ReSDS group was not significantly different from that of the S6 group, suggesting that the sensory-motor alterations induced by social density reduction may be reversible through resocialization (Fig. 4**D**).

**Fig. 4:**
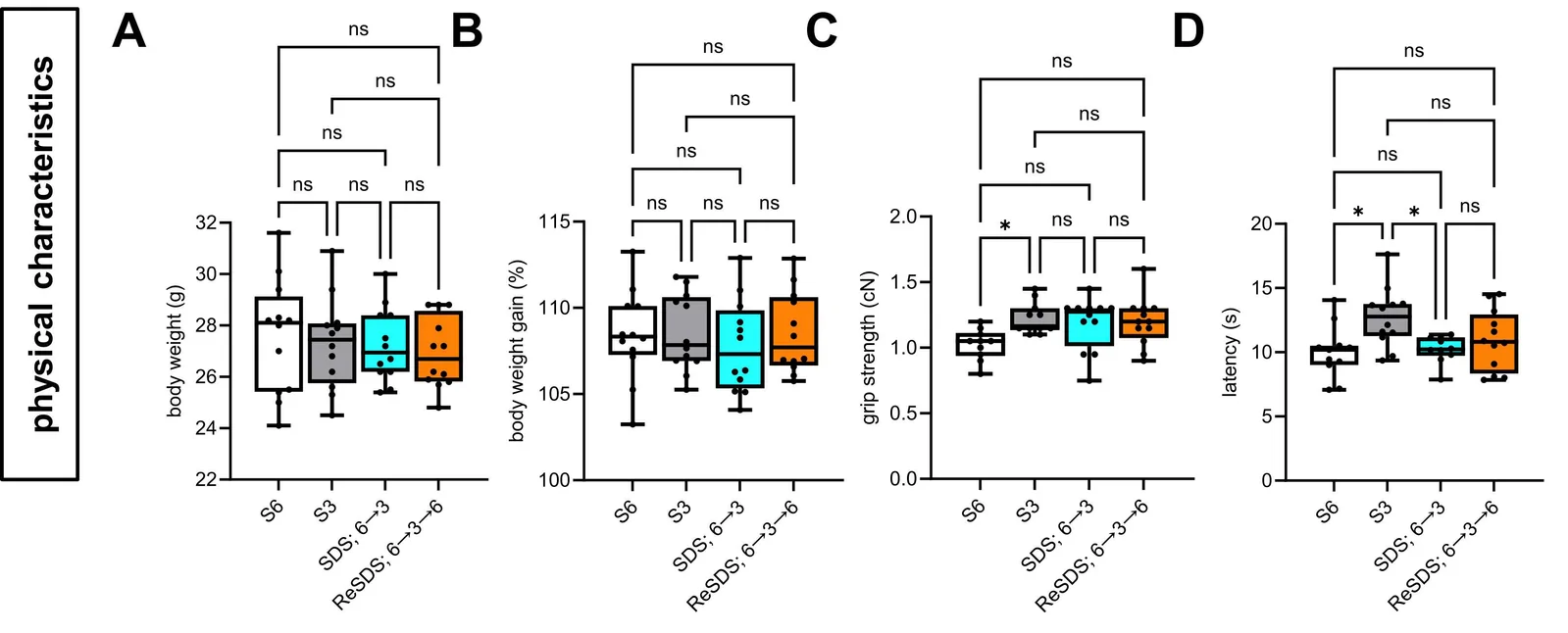
Effects of social downsizing and resocialization on physical characteristics and sensory response at 13 weeks of age. (A) Body weight at 13 weeks of age. (B) Body weight gain from 11 weeks to 13 weeks of age. (C) Grip strength. (D) Latency to response in the hot plate test. Data are presented as box-and-whisker plots. Statistical significance was determined by one-way ANOVA followed by Tukey’s post-hoc test. *p < 0.05; ns, not significant.

### Behavioral performance at 13 weeks of age and the effects of resocialization

To determine whether the behavioral changes induced by social downsizing stress (SDS) were reversible, we conducted a second battery of behavioral tests between 13 and 14 weeks of age. In the light/dark transition test, no significant differences were observed among the four experimental groups in total distance traveled (Fig. 5**A**), number of entries into the light compartment (Fig. 5**B**), time spent in the light area (Fig. 5**C**), or latency to the first entry into the light area (Fig. 5**D**).

**Fig. 5:**
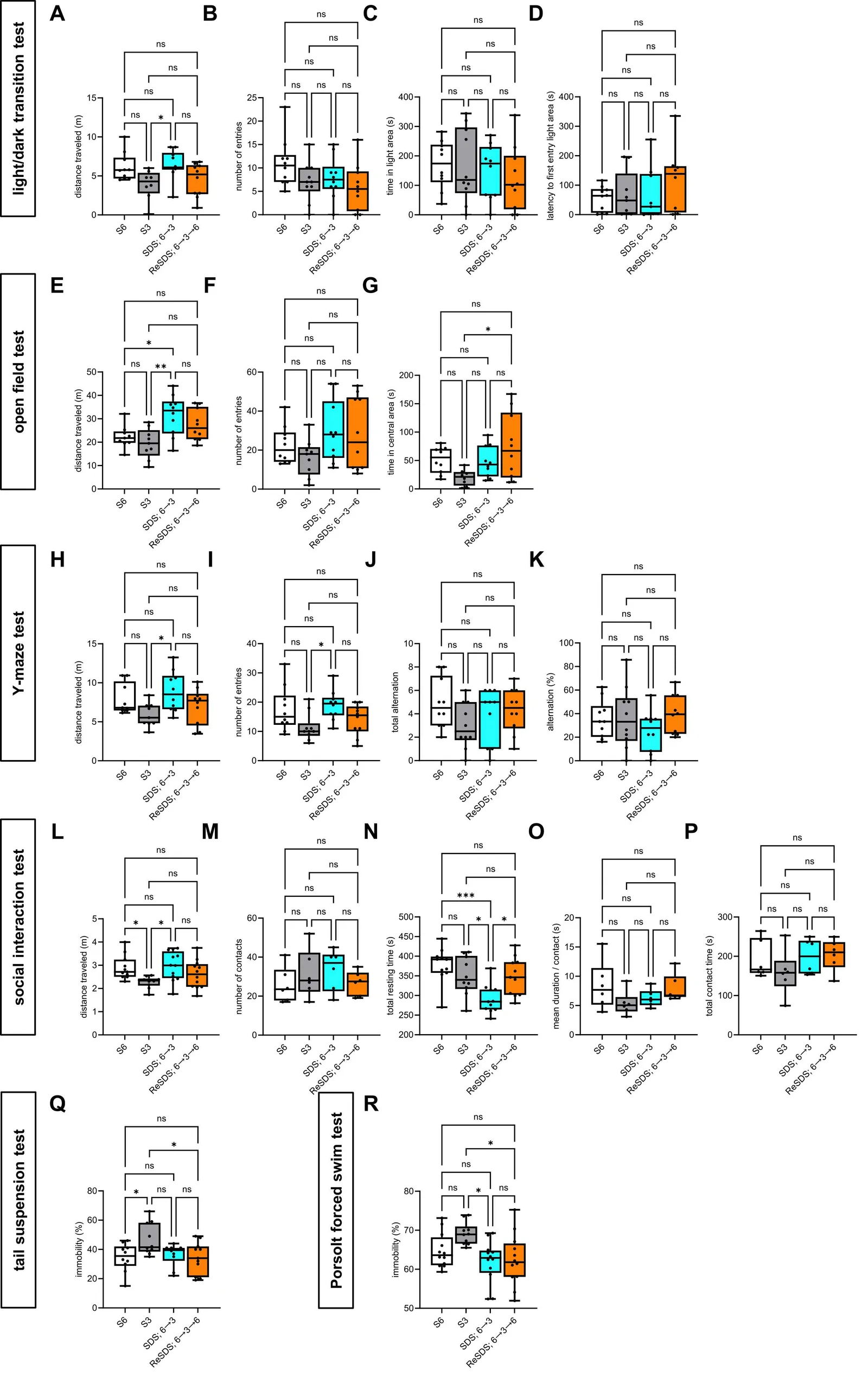
Effects of social downsizing and resocialization on behavioral phenotypes at 13 weeks of age. (A–D) Light/dark transition test: total distance traveled (A), number of entries into the light compartment (B), time spent in the light area (C), and latency to first entry into the light area (D). (E–G) Open field test: distance traveled (E), number of entries (F), and time spent in the central area (G). (H–K) Y-maze test: distance traveled (H), number of entries (I), total number of spontaneous alternations (J), and percentage of spontaneous alternation (K). (L–P) Social interaction test: distance traveled (L), number of contacts (M), total resting time (N), mean duration per contact (O), and total contact time (P). (Q) Tail suspension test: immobility percentage. (R) Porsolt forced swim test: immobility percentage. Data are presented as box-and-whisker plots. Statistical significance was determined by one-way ANOVA followed by Tukey’s post-hoc test. *p < 0.05, **p < 0.01, ***p < 0.001; ns, not significant.

In the open field test, the hyperactive phenotype observed at 10 weeks persisted in the groups that had experienced SDS. Both the SDS and ReSDS groups showed a significantly increased distance traveled compared to the S6 control group, indicating that resocialization did not mitigate the SDS-induced hyperactivity (Fig. 5**E**). Notably, the ReSDS group exhibited a significantly higher duration in the central area compared to the S3 group (Fig. 5**G**), suggesting that returning to a high-density environment can improve anxiety-like levels compared to chronic low-density housing.

Assessment of spatial working memory via the Y-maze test revealed that the deficits observed during the acute phase were no longer present. There were no statistically significant differences across any parameters, including distance traveled (Fig. 5**H**), number of entries (Fig. 5**I**), total alterations (Fig. 5**J**), or the percentage of spontaneous alternation (Fig. 5**K**), suggesting a spontaneous recovery of cognitive function by the chronic phase.

The social interaction test further highlighted the differential reversibility of SDS-induced behaviors. While the SDS group continued to display significantly increased locomotor activity (Fig. 5**L**) and significantly reduced total resting time compared to the S6 and S3 controls (Fig. 5**N**), resocialization effectively restored social resting behavior. Specifically, the ReSDS group showed a significant increase in resting time compared to the SDS group, returning to levels comparable to the S6 control (Fig. 5**N**). No significant differences were observed in the number of contacts (Fig. 5**M**), mean duration per contact (Fig. 5**O**), or total contact time among the groups (Fig. 5**P**).

## Discussion

The present study investigated the behavioral consequences of dynamic reductions in social group size using a newly established experimental paradigm termed Social Downsizing Stress (SDS). Unlike conventional rodent stress models that rely on complete social isolation or overt social conflict, the SDS paradigm selectively manipulates group size while maintaining social contact. Our results demonstrate that a transition from higher- to lower-density housing produces a distinct behavioral phenotype that differs from that observed under chronically low-density conditions (S3). Moreover, while several behavioral alterations induced by social downsizing—such as cognitive deficits and social resting patterns—were reversible following resocialization, others, specifically hyperactivity, persisted even after the restoration of the original social environment.

To clarify the specific contribution of the SDS paradigm, it is useful to position it explicitly relative to several established rodent social-environmental models. Social isolation paradigms, in which animals are housed individually from weaning or in adulthood, eliminate social contact entirely and are associated with well-characterized anxiety- and depression-like phenotypes together with HPA-axis and neuroinflammatory changes (Koolhaas et al., 2011). SDS differs fundamentally from this approach in that social contact is continuously maintained throughout the paradigm; only the number of cage-mates is reduced. Partial isolation paradigms, in which animals are housed alone for restricted periods (e.g., several hours per day), similarly interrupt social contact intermittently, whereas in SDS social contact is uninterrupted but permanently reduced in scale. Environmental impoverishment paradigms manipulate the physical and sensory complexity of the housing environment — for example, by removing nesting or enrichment materials or reducing cage space — rather than social composition; because no environmental enrichment was provided to any group in the present study and cage size was held constant across groups, SDS isolates the social dimension of the manipulation from physical environmental impoverishment. Crowding stress paradigms move in the opposite direction, increasing group density beyond standard housing conditions and typically eliciting increased territorial competition and agonistic behavior; SDS instead reduces density from a standard baseline, representing a mechanistically distinct manipulation. Finally, social instability paradigms, most extensively characterized in female mice, involve repeated shuffling of cage composition with unfamiliar conspecifics over consecutive days, modeling unpredictability and repeated relationship disruption (Jarcho et al., 2016). SDS differs from this approach in that it involves a single, one-time reduction in group size among animals that remain familiar to one another throughout, without the introduction of unfamiliar conspecifics or repeated social disruption. Taken together, SDS is distinguished from these established paradigms by its specific combination of features: a single, permanent reduction in social group size, implemented among already-familiar animals, with social contact continuously maintained and physical housing conditions held constant. We acknowledge, however, that direct head-to-head behavioral or physiological comparisons between SDS and these established paradigms were not performed in the present study, and such comparisons represent an important direction for future work to further establish the unique contribution of this model.

In the acute phase following group-size reduction (10 weeks of age), SDS mice displayed what we operationally define as a "hyper-reactive" behavioral profile: a coordinated, multi-domain pattern combining (i) increased overall locomotor activity in the open field, (ii) increased approach behavior toward an aversive/illuminated compartment in the light/dark transition test, (iii) reduced occupancy of the anxiogenic central zone of the open field, and (iv) reduced social engagement (decreased social resting time), occurring together rather than any single measure changing in isolation. We use this term specifically to distinguish the present phenotype from simple hyperactivity, which we take to refer narrowly to increased locomotor output alone (e.g., distance traveled), and from generalized increased exploratory behavior, which would be expected to increase, rather than decrease, time spent in anxiogenic areas such as the central zone of the open field. In the present data, increased approach behavior in the light/dark test co-occurred with reduced central-zone exploration in the open field, indicating that the SDS phenotype does not reflect a uniform increase in exploration or approach behavior across all anxiogenic contexts, but rather a specific, context-dependent pattern of altered reactivity. Specifically, the SDS group showed significantly increased locomotor activity in the open field (OF) and a higher number of entries in the light/dark (LD) transition test. Importantly, these behavioral changes were not observed in mice maintained under stable low-density housing (S3), indicating that the transition itself, rather than low social density per se, contributed to the observed behavioral phenotype. This distinction is critical because most existing studies have focused on static environmental conditions, whereas real-world social environments frequently involve dynamic transitions. The present findings therefore suggest that behavioral responses to social environmental change depend not only on the absolute level of social density but also on the history of social experience (Branchi et al., 2006, Lukkes et al., 2009).

Theoretical frameworks in stress biology offer possible, though as yet untested, explanations for these effects. Changes in group size can alter the frequency of social interactions, disrupt established social hierarchies, and modify environmental predictability (McEwen, 2007), and disruptions of this kind have been hypothesized, in other contexts, to trigger adaptive stress responses that recalibrate behavioral strategies in response to perceived environmental instability. In addition, the concept of social buffering suggests that social partners can mitigate physiological and behavioral responses to stress in other paradigms (Hennessy et al., 2009, Kikusui et al., 2006); by extension, a reduction in group size from six to three mice could plausibly weaken such buffering mechanisms. However, we did not directly assess stress-responsive neural or endocrine systems in the present study, and any link between social buffering and the regulation of these systems in the SDS paradigm remains hypothetical. The behavioral alterations we did observe across multiple domains — including impaired spatial working memory in the Y-maze and reduced resting time during social interaction — are broadly compatible with, but do not themselves establish, an underlying stress- or buffering-related mechanism of this kind.

A related and important consideration is whether the observed SDS phenotype reflects the loss of social density per se, or a reorganization of social hierarchy triggered by the reduction in group size. Reducing a cage from six to three animals inevitably alters dominance relationships, territorial dynamics, and social competition, and these factors could plausibly contribute to the behavioral changes observed in the SDS group. In the present study, the division of each six-mouse cage into two three-mouse subgroups was performed randomly with respect to pre-existing dominance status, and each resulting subgroup consisted exclusively of animals that had previously been housed together; this design minimizes, but does not eliminate, the possibility that pre-established hierarchical relationships selectively determined subgroup composition. However, we did not directly assess dominance hierarchies, territorial behavior, or agonistic interactions (e.g., using the tube test, resident–intruder paradigm, or quantification of barbering/wound patterns) either before or after the downsizing procedure. It therefore remains possible that the abrupt reduction in group size triggered a period of hierarchy reorganization or heightened social competition within the newly formed three-mouse groups, and that this process — rather than, or in addition to, the reduction in social density itself — contributed to the observed hyper-reactive phenotype. Notably, this interpretation is not necessarily exclusive of the reduced-density hypothesis, given that the stable S3 group, which did not undergo an equivalent transition, did not display the same behavioral profile as the SDS group. Nevertheless, directly disentangling "loss of social density" from "reorganization of social hierarchy" will require future studies incorporating explicit measures of dominance and social structure before and after downsizing.

Interestingly, several behavioral alterations induced by SDS were reversible when the original social group size was restored. At 13–14 weeks of age, resocialization (ReSDS) normalized spatial working memory performance and social resting behavior, suggesting that these behavioral domains retain substantial plasticity even after exposure to social environmental perturbation. These findings align with previous work demonstrating that social experience can dynamically modulate neural and behavioral systems throughout development and adulthood (Branchi, 2009). It should be noted, however, that normalized Y-maze alternation performance does not necessarily indicate a full restoration of underlying cognitive processes. Spontaneous alternation is sensitive not only to spatial working memory but also to locomotor activity, exploratory motivation, and anxiety-like state, any of which could secondarily influence alternation behavior independent of true mnemonic function. Notably, within the Y-maze apparatus itself, distance traveled and the number of arm entries did not differ significantly between groups at either the acute (Fig. 3**H, I**) or chronic (Fig. 5**H, I**) timepoint, despite clear group differences in open-field locomotor activity at the same timepoints (Figs. 3**E,** 5**E**). This dissociation suggests that the acute Y-maze impairment in the S3 and SDS groups, and its subsequent normalization in the ReSDS group, is unlikely to be a simple by-product of generalized hyperactivity within the testing apparatus. Nevertheless, because anxiety-like behavior also differed between groups at 10 weeks of age (Fig. 3**E–G**), a contribution of altered motivation or anxiety-related exploratory strategy to the observed Y-maze phenotype cannot be excluded. Future studies employing complementary, less activity-dependent measures of spatial working memory (e.g., the novel object recognition test, radial arm maze with fixed trial number, or T-maze delayed alternation with controlled inter-trial intervals) would help to clarify whether the SDS/S3-induced impairment and its resocialization-dependent recovery reflect genuine changes in cognitive function, as opposed to secondary effects of locomotor or affective state."

In contrast, the hyperactivity observed in the open field test persisted even after resocialization, indicating that this aspect of the SDS-induced phenotype is more enduring than the reversible changes described above. We propose that the experience of losing social density may produce long-lasting changes in behavioral arousal or environmental vigilance; consistent with Social Baseline Theory, a reduction in social group size may shift the baseline that social environments provide for regulating perceived risk and behavioral effort (Coan and Sbarra, 2015). However, alternative, non-mutually-exclusive explanations cannot be excluded on the basis of the present data, including differences in habituation, altered exploratory drive, changes in social hierarchy following group-size reduction, or generalized shifts in arousal that are unrelated to a lasting "scar." Because hyperactivity was assessed only up to 13–14 weeks of age using independent cohorts at each time point, the present design demonstrates persistence of this phenotype within the tested window but does not establish permanence or true irreversibility. Longitudinal, within-subject tracking beyond 14 weeks, together with supporting biological evidence, will be required to determine whether this represents a stable, long-term "behavioral scar" or a more transient, slowly resolving alteration. Similar long-lasting behavioral consequences have nonetheless been reported in other rodent models of early or chronic social stress, including social defeat and social isolation paradigms (Krishnan et al., 2007, Golden et al., 2011).

The present study has several limitations. First, the experiments were conducted exclusively in male mice, and sex differences in responses to social environmental change were not examined. Previous research suggests that social stress responses can differ significantly between males and females (Bangasser and Valentino, 2014, Jarcho et al., 2016), highlighting the importance of evaluating both sexes in future studies. Second, all outcomes reported in this study are behavioral in nature; no molecular, endocrine, electrophysiological, neuroimaging, or histological measurements were obtained. In particular, no physiological or neuroendocrine indices of stress (e.g., plasma corticosterone, ACTH, HPA-axis activity, autonomic measures) were assessed. Accordingly, the term "stress" in the SDS paradigm is used in an operational, behaviorally defined sense, and the neurobiological basis of the SDS phenotype — including any underlying molecular, endocrine, electrophysiological, structural, or histological correlates — remains entirely unknown at present. Future studies incorporating such measures will be essential to establish the biological basis of the SDS phenotype and to more rigorously justify its classification as a stress model, as distinct from a purely behavioral social-transition paradigm. Third, this study did not directly assess social hierarchy or dominance relationships within cages before or after the downsizing procedure. Although the division of each six-mouse cage into two three-mouse subgroups was performed randomly with respect to pre-existing dominance status, we cannot fully exclude the possibility that hierarchy reorganization or increased social competition, rather than the reduction in social density itself, contributed to the observed behavioral phenotype. Future studies incorporating dominance assays (e.g., the tube test) or continuous monitoring of agonistic behavior before and after downsizing will be needed to clarify this potential confound. Future studies are necessary to clarify the biological substrates of SDS-induced behavioral changes. Fourth, the SDS manipulation was applied at a single developmental time point (8 weeks of age). Because the transition from adolescence to young adulthood is a highly sensitive period for social and neurobehavioral development in rodents, the specific timing of social downsizing may significantly influence the resulting phenotype, and the present findings should not be generalized to downsizing occurring at earlier (e.g., peri-weaning) or later (fully adult) developmental stages. Relatedly, the approximately two-week interval preceding the first behavioral assessment — extended relative to the one-week interval used in our previous study (Tanaka et al., 2026) — was not derived from an explicit pilot comparison of multiple intervals within the present paradigm, and it remains possible that a different interval would yield a different behavioral profile. Future studies systematically comparing the effects of downsizing at multiple developmental time points will be needed to determine the extent to which developmental timing modulates the SDS phenotype. Fifth, mice underwent a battery of six (10 weeks) or eight (13–14 weeks) behavioral tests administered on consecutive days, one test per day, rather than a single behavioral domain assessed in isolation. Although tests were ordered from least to most aversive and separated by 24-h intervals to minimize carryover effects, we cannot fully exclude the possibility that prior testing experience influenced performance on subsequent tests through habituation, repeated stress exposure, or learning effects. This is a common limitation of comprehensive behavioral batteries and should be considered when interpreting the present findings.

Despite these limitations, the SDS paradigm provides a novel experimental framework for investigating the neurobehavioral consequences of social environmental transitions. By isolating the effects of dynamic changes in group size from those of chronic low-density housing or complete social isolation, this model offers new opportunities to study how social structure shapes behavioral adaptation. We note that human reductions in social networks — for example, through changing household composition — involve emotional, cognitive, cultural, and economic dimensions that are not represented in this cage-density manipulation, and the SDS paradigm should not be interpreted as a direct model of these human phenomena. Rather, by modeling one isolable structural component of such transitions, this paradigm may offer a starting point for future, more cautious inquiry into how quantitative changes in social scale relate to behavioral and neural adaptation, with potential long-term relevance to mental health research.

## Conclusion

Social downsizing during young adulthood induces a distinct behavioral phenotype in mice, characterized by hyper-reactivity that differs from that observed under static low-density housing, highlighting the critical impact of social transition history on behavioral adaptation. While cognitive deficits and social resting patterns are reversible through resocialization, hyperactivity persisted throughout the tested period regardless of environmental restoration, suggesting a potential long-lasting behavioral scar; however, longitudinal within-subject data and biological evidence will be needed to establish true irreversibility. As these findings are based solely on behavioral outcomes, future studies incorporating physiological and neuroendocrine measures will also be needed to determine whether the SDS paradigm engages canonical stress pathways. Nonetheless, this model provides a novel framework for understanding the enduring neurobehavioral consequences of shrinking social scales in modern society. Given the structural, cage-based nature of this manipulation, direct extrapolation to the emotional and socioeconomic complexity of human social network changes should be made with caution.

## Ethics declaration

This study was conducted in accordance with the ARRIVE (Animal Research: Reporting of In Vivo Experiments) guidelines. This study was approved by the Committee for Animal Experiments of Kawasaki Medical School.

## Ethics approval and consent to participate

All animal experiments were conducted in accordance with relevant guidelines and regulations and adhered to the ARRIVE guidelines (https://www.nc3rs.org.uk/arrive-guidelines) and the U.S. National Institutes of Health *Guide for the Care and Use of Laboratory Animals* (8th edition, 2011). The study was approved by the Committee for Animal Experiments of Kawasaki Medical School. Every effort was made to minimize the number of animals used and to reduce their suffering. The experimental design aimed to ensure scientific rigor and statistical validity while using the smallest feasible number of animals per group and limiting the total number of experimental groups.

## Funding

This study did not receive any specific grant from funding agencies in the public, commercial, or not-for-profit sectors. The authors also declare that no funding source had any role in the study design, data collection, analysis, interpretation, or writing of the manuscript.

## CRediT authorship contribution statement

**Yoshihiro Tanaka:** Supervision, Data curation. **Hiroshi Ueno:** Writing – original draft, Investigation, Data curation, Conceptualization. **Eriko Kitano:** Supervision. **Yu Takahashi:** Supervision. **Sachiko Mori:** Supervision. **Shinji Murakami:** Supervision. **Kenta Wani:** Supervision. **Yosuke Matsumoto:** Supervision. **Motoi Okamoto:** Writing – review & editing, Supervision, Project administration. **Takeshi Ishihara:** Writing – review & editing, Supervision, Project administration, Conceptualization.

## Declaration of Generative AI and AI-assisted technologies in the writing process

During the preparation of this work, the authors used ChatGPT Plus (OpenAI) and Gemini (Google) to assist with language refinement and content organization. The authors subsequently reviewed and edited the content as needed and take full responsibility for the final version of the manuscript.

## Declaration of Competing Interest

None

## Footnotes

Appendix A Supplementary data associated with this article can be found in the online version at doi:10.1016/j.ibneur.2026.10.003.
